# Recurrent Syncope Associated with Lung Cancer

**DOI:** 10.1155/2015/309784

**Published:** 2015-05-12

**Authors:** Dingguo Zhang, Liansheng Wang, Zhijian Yang

**Affiliations:** Department of Cardiology, The First Affiliated Hospital of Nanjing Medical University, No. 300, Guangzhou Road, Nanjing, Jiangsu 210029, China

## Abstract

Syncope is an important problem in clinical practice with many possible causes that might be misdiagnosed. We present an unusual case of syncope, which has a normal chest X-ray. Exercise EKG and coronary angioplasty results confirmed the existence of serious coronary heart disease. The patient was treated with coronary stent transplantation. However, scope occurred again and the elevated tumor makers cytokeratin-19-fragment and neuron-specific enolase revealed the bronchogenic carcinoma, which was confirmed by enhanced CT examination. The treatment of carcinoma by chemotherapy was indeed sufficient for prompt elimination of the syncope symptoms.

## 1. Introduction

Syncope is an important problem in clinical practice, with a frequency between 15% and 39%; however, even with advanced technology, nearly one-third of cases reported no certain reason of syncope being found. The causes of syncope include cardiac, neurologic-cerebrovascular, vascular, psychogenic and metaboli-miscellaneous, but some uncommon causes should be kept in mind to avoid misdiagnosis [1]. We present an unusual case of syncope, which was firstly attributed to coronary heart disease and treated with coronary stent transplantation. The chest X-ray of this patient was normal; however, the causative factor, bronchogenic carcinoma, was diagnosis unanticipated by the hint of elevated tumor maker cytokeratin-19-fragment (CYFRA21-1) and neuron-specific enolase (NSE).

## 2. Case Report

**Figure 1:**
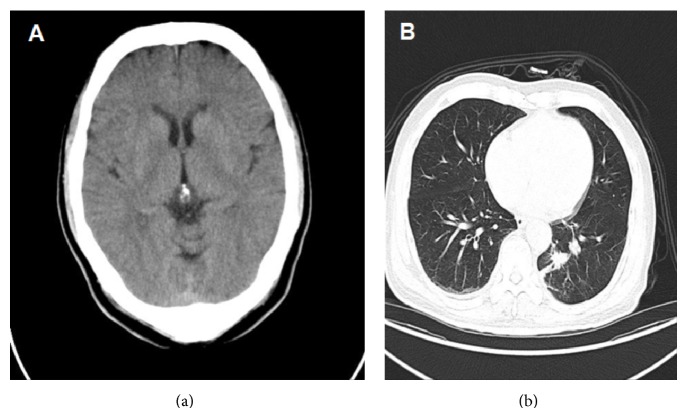
(a) Cranial CT produced no unusual findings. (b) Enhanced chest CT showed a mass with irregular border in the left to middle mediastinum.

A 64-year-old man, a 30-year-long cigarette smoker (30 pieces per day), was admitted with syncope twice on one day to our hospital emergency department in Oct 17, 2013. The patient has two years of high blood pressure history while without any medicine treatment. He had no diabetes mellitus history. In the past two months, he felt chest discomfort sometimes. In the morning of Oct 14, 2013, he suddenly lost consciousness with incontinence while walking in the park. He felt pericardium discomfort before losing consciousness. After regaining consciousness several minutes later, he felt dyspnoeic and sweaty. In the afternoon, syncope occurred again while he was watching TV. His electrocardiogram showed a slight sinus bradycardia of 47 bpm. 24-hour Holter indicated the average heart rate was 66 bpm, the slowest heart rate 47 bpm, the fastest heart rate 129 bpm, and the longest R-R interval 1.6 second. Cranial CT produced no unusual findings (Figure 1(a)). Moderate ST-segment depression was determined in lead II and aVF on treadmill exercise ECG. Ultrasound cardiography (UCG) indicated normal function with the left ventricular ejection fraction 69.4%. No unusual finding was found in chest X-ray. Blood myocardial enzyme, renal function, electrolytes, and D-dimer values were all within normal ranges.

Five days later, the patient had coronary angiography examination. Coronary angiography revealed 80% stenosis of proximal segment and 85% stenosis of distal segment in left anterior descending (LAD) coronary artery, 90% stenosis of middle and distal segment in left circumflex (LCX) coronary artery, 50% stenosis of proximal segment, and 80% stenosis of middle segment in right coronary artery (RCA). The patient refused to have coronary artery bypass grafting surgery. So we decided to perform percutaneous coronary intervention (PCI). After balloon predilatation, 3.0 × 24 mm EXCEL rapamycin-eluting stent (Jiwei, Shandong, China) was implanted in RCA. A 2.5 × 14 mm EXCEL rapamycin-eluting stent and a 3.0 × 21 mm Partner sirolimus-eluting stent (Raisedragon, Peking, China) were implanted in distal and proximal segment of LAD, respectively. The further PCI was scheduled one week later for LCX. Unexpectedly, syncope occurred again. On the afternoon while sitting 2 days later, symptoms of dyspnea and palpation accompanied with hypotension occurred. Electrocardiogram monitor indicated that the heart rate was serious sinus bradycardia of nearly 30 bmp. Blood pressure was below 80/40 mmHg. The symptom disappeared after atropine and dopamine were infused. Consider the patient is a 64-year-old person with 30-year-long cigarettes intake history. We put a serum marker screen. Unexpectedly serum tumor marker results indicated that carbohydrate antigen 19-9, carbohydrate antigen 72-4, and alpha fetoprotein are all within normal ranges whereas cytokeratin-19-fragment (CYFRA21-1) and neuron-specific enolase (NSE) values elevated slightly (CYFRA21-1 3.75 ng/mL, normal <3.3 ng/mL; NSE 16.62 ng/mL, normal <16.3 ng/mL).

Examination of enhanced chest CT led to suspicion of central lung cancer (Figure 1(b)). PET-CT confirmed the leaf of central type lung cancer (4.2 × 3.7 × 2.6 cm) of left lung with multiple lymph node metastasis and the tumor infiltration of adjacent thoracic aorta and left pulmonary artery and vein. No brain metastasis or heart metastasis was found on PET-CT examination. Immunohistochemical examination of the specimens collected with bronchoscopy revealed the following: CKpan(+), CKL(+), Syn(+), CgA(+), CD50(+), CD30(−), CK20(−), CK7(−), CK5/6(−), P63(−), and Ki-67(+).

The diagnosis of small cell bronchogenic carcinoma was made; this tumor was clinically staged at T4N2M0 IIIB. Chemotherapy was promptly initiated with use of combination of carboplatin and topotecan. Shortly after the first chemotherapeutic infusion, the patient reported feeling much better. At follow-up a year later, enhanced CT scan showed that the tumour volume decreased and the patient did not experience syncope anymore.

## 3. Discussion

Syncope is the reason for one to three percent of visits to emergency departments and admissions to hospital, affecting about three to six out of every thousand people each year. The risk of a bad outcome, however, depends very much on the underlying cause [2]. However, some uncommon causes should be kept in mind to avoid misdiagnosis.

Accounting for 10% to 20% of cases of syncope, a cardiac cause is the main concern in patients presenting with syncope, as cardiac syncope predicts an increased risk of death and may herald sudden cardiac death [2]. It often occurs suddenly without any warning signs, in which case it is called malignant syncope [3]. Unlike what occurs in neurally mediated syncope, the postrecovery period is not usually marked by lingering malaise. Cardiac syncope is often due to structural heart disease with cardiac obstruction, ventricular tachycardia, or bradyarrhythmias. The interest in the present clinical case, especially for the cardiologic community, is the fact that the patient initially presented with a medical history typical of cardiac syncope. The patient has the chest discomfort history for 2 months before syncope occurred. At the same time, results of the treadmill exercise ECG were suggestive of coronary artery disease and coronary angiography examination demonstrated multiple coronary lesions. Therefore, the main consideration for the syncope diagnosis is due to the coronary heart disease. However, after the coronary stent transplantation in RCA and LAD coronary artery, syncope occurred again. Lung cancer is a disease of global geographic reach that is nondiscriminatory in attacking all people, regardless of age, sex, ethnicity, or socioeconomic background [4]. This disease is often suspected on the basis of presenting symptoms and signs. The most frequent symptoms are cough, wheeze, dyspnoea, and haemoptysis. However, the presentation of lung cancer varied quietly. Most people have no sign at first and until the end stage, the disease is noticed and diagnosed but it is too late to treat. So adults of age 55 to 80 years who have a 30 pack-year smoking history and are currently smoking or have quit within the past 15 years are recommended screening with low-dose computed tomography [5]. The chest X-ray is normal, and the patient has no sign of respiratory tissue disease. Therefore, at the first time, we could not consider the lung disease as the possible cause of syncope.

Very interestingly, the unexpected oncologic markers CYFRA21-1 and NSE values elevated slightly, which gave us the hint that tumor could not be excluded. Serum tumor markers are considered as biological indicators detected from the serum or plasma of suspected tumor patients with insufficient sensitivity or specificity [6], which possess the advantages of easy detection, noninvasive operation, and cost-effectiveness [6, 7]. For years, the oncologic literature suggests that serum CYFRA21-1 and NSE can be a potential serologic biomarker in evaluation of lung cancer, especially small cell lung cancer (SCLC) [8]. Although the chest X-ray was normal, we put the further imaginative examination with enhanced chest CT and PET-CT, which confirmed the suspicious diagnosis of lung cancer. And further immunohistochemical examination of the specimens collected with bronchoscopy indicated the diagnosis of SCLC. In the past decades, many types of cancer associated syncopes have been reported [9–11], especially when a tumor mass invades the baroreceptor within the carotid sinus or when it disrupts the afferent nerve fibers of the glossopharyngeal nerve. However, only little cases of syncope induced by lung cancer were reported [12]. To the best of our knowledge, this is the first report of lung cancer associated syncope diagnosis by the hint of elevated tumor marker. On PET-CT examination, no evidence of brain metastasis or heart metastasis was found. Therefore, we think that paraneoplastic symptoms might be the cause for this patient's syncope although we did not test the Hu, anti-Yo, or other antibodies. Paraneoplastic syndromes are rare disorders that are triggered by an altered immune system response to a neoplasm, which might be the first or most prominent manifestation of a cancer, specifically small cell carcinomas of the lung [13]. In a broad sense, these syndromes are collections of symptoms that result from substances produced by the tumor, and they occur remotely from the tumor itself. The symptoms may be cardiovascular, endocrine, neuromuscular or musculoskeletal, cutaneous, hematologic, gastrointestinal, renal, or miscellaneous in nature. The relationship between small cell lung cancer and the neurocardiogenic syncope in our case is suggested by the closed temporal sequence and almost immediate improvement after chemotherapy, which is similar to the findings in the case previously reported [14].

In view of such experience, we believe that an atypical medical history of newly developed syncope in an elder patient should alert the cardiologist to the possibility of lung cancer. Syncope is an important problem in clinical practice. Obtaining a detailed history combining all clinical and laboratory findings is very important, particularly in elderly patients who exhibit multiple risk factors for several diseases. Although syncope occurs commonly, lung cancer especially in the context of serious coronary heart disease occurring simultaneously is extremely rare. Such misleading manifestations require a high index of suspicion on behalf of the physician, so that an underlying malignancy is not missed, and a final diagnosis combining all clinical and laboratory findings is reached. In turn, in rare cases common tumor markers such as CYFRA21-1 and NSE can be used as a useful tool driving further management. The association of abnormal coronary motility, neurocardiogenic syndrome, and lung carcinoma seems to be new in the literature and may suggest a wider spectrum of the paraneoplastic neuropathies. Possibly, some peculiar clinical features and surely a unique treatment (chemotherapy) seem to characterize and cease the cancer associated neurocardiogenic syncope. Syncope is an important problem in clinical practice.
